# Predictive processing and the representation wars: a victory for the eliminativist (via fictionalism)

**DOI:** 10.1007/s11229-017-1442-8

**Published:** 2017-05-26

**Authors:** Adrian Downey

**Affiliations:** Department of Philosophy, University of Sussex, Brighton, UK

**Keywords:** Sub-personal representation, Eliminativism, Fictionalism, Predictive processing

## Abstract

In this paper I argue that, by combining eliminativist and fictionalist approaches toward the sub-personal representational posits of predictive processing, we arrive at an empirically robust and yet metaphysically innocuous cognitive scientific framework. I begin the paper by providing a non-representational account of the five key posits of predictive processing (“prediction-signal”, “error-signal”, “prior”, “likelihood”, and “posterior probability”). Then, I motivate a fictionalist approach toward the remaining indispensable representational posits of predictive processing, and explain how representation can play an epistemologically indispensable role within predictive processing explanations without thereby requiring that representation metaphysically exists. Finally, I outline four consequences of accepting this approach and explain why they are beneficial: (1) we arrive at a victory for metaphysical eliminativism in the ‘representation wars’; (2) my account fits with extant empirical practice; (3) my account provides guidance for future research; and, (4) my account provides the beginnings of a response to Mark Sprevak’s IBE problem for fictionalist approaches toward sub-personal representation.

## Introduction

Predictive processing is a rapidly developing framework which is on course to become the dominant research paradigm in cognitive science. One of the chief philosophical exponents of this framework, Andy Clark, has recently claimed that predictive processing resolves the debates surrounding the concept “representation” and its relevance to work in cognitive science. According to Clark:

[W]e have here moved so far from a once-standard complex of ideas concerning the nature and role of the inner states underlying perception and action that stale old debates concerning the existence, nature, and role of ‘internal representations’ should now be abandoned and peace declared. (2015a, p. 1).

In this paper I am going to concur with Clark that predictive processing does require a cessation of hostilities in the ‘representation wars’.^1^ However, contra Clark, I will argue that this cessation is required because the anti-representational camp has won a decisive victory (in the predictive processing theatre)—the representational posits of predictive processing can either be eliminated completely, or they should be assigned a fictional status.

The paper itself is structured as follows—in Sect. 2 I outline predictive processing and explain how its key posits can be accounted for without requiring representation. In Sect. 3, I provide a fictionalist account of predictive processing’s remaining indispensable representational posits. Finally, in Sect. 4, I highlight four benefits one accrues by accepting this approach.

## Predictive processing and representation

On the predictive processing (PP) framework, the brain is considered to be an inference machine which uses approximations of Bayesian probability theory to maintain its own viability. PP explains action and perception to be driven by one simple tenet: minimise prediction error. Prediction error-signals signal that a given prediction is false—the sensory signal received by the brain was not the sensory signal which it predicted would be received. By minimising prediction error, the brain should be led to situations which allow it to maintain itself within prescribed homeostatic boundaries. PP understands perception to be constituted by the brain’s ‘best guess’ about the ultimate causes of its current sensory states. Importantly, this ‘best guess’ is thought to be fundamentally *action-oriented *because it pertains to the perception of environmental *affordances *(Clark 2016; Friston 2012; Wiese and Metzinger 2017; *cf*. Gibson 1979). Furthermore, because PP is advanced within the *dynamic *tradition of cognitive science, cognition is considered to come as part of the ‘package deal’ once action and perception have been explained (Clark 2016; Hurley 2001). Therefore, PP explains perception, action, and cognition to all be executed via prediction-error minimisation.

The brain’s ‘best inference’ will be the hypothesis which has the highest overall *posterior probability. *Posterior probability is the overall probability assigned to a hypothesis (given relevant information) and it is calculated by multiplying the hypothesis’ prior probability and its likelihood, and then dividing this amount by the prior probability that the information on which these calculations are based is correct. The prior probability is the probability given to the hypothesis independently of current events. For example, the prior probability of my being perceptually presented with a cat is quite high, because there are many cats living in my street. The prior probability of my being presented with a leopard, however, is quite low. Leopards are rarely, if ever, to be found in my street. The likelihood of a hypothesis can be calculated as follows—assume the truth of the hypothesis in question, and then determine the probability of the evidence actually acquired being collected if that hypothesis were true. For example, if I encounter a cat in my street then the hypothesis “I am perceiving a cat” will be accorded a high likelihood because my sensory stimulation is highly probable in the context of this hypothesis. Similarly, if I happen to confront an escaped leopard in my street, the hypothesis “there is a leopard in front of me” would be accorded a high likelihood. In both cases, the hypothesis in question is highly likely because the sensory stimulation received is highly probable given the hypothesis.^2^

Having outlined the key posits of PP (“prediction-signal”, “error-signal”, “posterior probability”, “prior”, and “likelihood”) I am now going to explain how each can be satisfactorily explained without invoking the concept “representation”. I will do so by drawing on arguments in this vein recently proffered by Orlandi (2015; cf. 2014) and explicitly linking them in with William Ramsey’s *job-description challenge*. I will explain why these PP posits do not meet the job-description challenge, and so conclude that the key posits of PP can be accepted without thereby requiring adoption of representation.

### Ramsey’s “job-description challenge”

When it is used in cognitive science, the concept “representation” is considered to be a psychological notion which requires the invocation of properties such as “aboutness”, “truth-conditions”, and “content”. These properties are not typically thought to exist in non-cognitive physical entities, such as stones or trees. As such, they are to be considered special, higher-level properties, which are not ubiquitous in the natural world. Accordingly, we must have good reasons to describe a given cognitive mechanism or system in terms of representation.

William Ramsey has recently argued that we can only ascribe “representation” to a cognitive mechanism or system if it passes “the job-description challenge” (2009). If a mechanism or system is to pass this challenge it must: (1) play a functional role within the cognitive system which we would pre-theoretically understand to be representational in nature; and, (2) it must be explanatorily beneficial to treat the mechanism as functioning in this manner. Ramsey argues that condition (1) must be met because, otherwise, the concept of “representation” would become empirically vacuous. If we re-define the concept “representation”, such that our use of the concept within cognitive science has nothing in common with our pre-theoretical psychological notion, then ascription of this concept would become meaningless. If condition (1) is not met, the “representational theory of mind” becomes a representational theory in name only. Condition (2) must be met, according to Ramsey, because otherwise the concept “representation” would become explanatorily vacuous. We can trivially describe any mechanism or system in terms of representation. For example, I can describe a stone rolling down a hill in terms of the stone desiring that it reaches the bottom, believing that rolling is the best way to achieve this aim, and so on. However, we do not receive any additional explanatory benefit from this representational account of stone rolling over and above that received from applying a purely non-representational, physicist’s account to the stone’s rolling behaviour. Thus, condition (2) must be met in order to avoid a complete trivialisation of the concept “representation”. Representation should only be ascribed to a cognitive mechanism if ascribing representation helps one gain a better understanding of that mechanism.

In short, “representation” (as it is used in cognitive science) is a psychological concept which is not ubiquitous throughout physical systems in nature. If we are to maintain a robust and empirically useful notion of representation then, according to Ramsey, a given cognitive mechanism should only be described in terms of representation if it deserves to be described in such terms. He suggests that we assess whether a mechanism does deserve to be described in terms of representation by submitting it to the job-description challenge. Ramsey argues that a cognitive mechanism should be described in terms of representation only if it passes this challenge.

### “Prediction” and “error” signals

Let us now submit the PP concepts of “prediction-signal” and “error-signal” to the job-description challenge. Both of these concepts appear prima-facie to require representation—predictions signal *that* such-and-such is the case, whilst errors signal *that* such-and-such is not the case. Nico Orlandi argues, however, that closer inspection of the role these terms play in PP explanations reveals they are not representational posits (2015; *cf.*^2014^). They fail the job-description challenge.

Predictions are present at all levels of the perceptual hierarchy and are passed down to the level immediately below their level of origin. Error-signals, on the other hand, are passed up from their level of origin to the level immediately above it. As such, each is concerned only with *proximal* conditions.^3^ Therefore, the signals being passed up and down the perceptual hierarchy are better understood in terms of causal covariation or correlation. In order to argue that these signals are representational in nature, one must therefore explain how or why brain-based causal covariation results in or requires representation. William Ramsey argues that accounts of brain-based representation founded upon causal covariation fail the job-description challenge, and so he concludes that mechanisms in the brain which function on the basis of causal covariation should not be considered representational (2009, ch. 4). His summary of this conclusion is worth quoting in full:

Despite its common appeal, the receptor notion of representation [Ramsey’s name for covariation based accounts] comes with a job description that, in this context, has little to do with the role of representation...When we look at the role of receptors *inside* of cognitive systems, as described by cognitive theories that employ them, we see that the role is better described as something like a reliable causal mediator or relay circuit which, as such, is not representational in nature. In other words, when a causal/physical system (like the brain) is described as performing various cognitive tasks by employing a structure that has the job of causing something to occur when and only when something else occurs, then the system is not, on the basis of this description alone, employing internal representations. (Ramsey 2009, p. 149, *italics in original*).

Ramsey’s argument, in essence, is that we gain no extra explanatory purchase by treating causal covariation within the brain in terms of representation. We do not arrive at better accounts of neural processing by giving causal covariation between neurons a representational status, because treating them as such does not provide one with any extra explanatory benefits over those one would accrue by treating them as mere non-representational causal correlations. Furthermore, treating causal covariation in terms of representation violates our pre-theoretic use of the concept. Therefore, causal correlation between neural processes fails the job-description challenge and so does not deserve a representational status.

The proponent of representation is likely to object, however, along the following lines:

The universe is stuffed with correlations and it is implausible to count them all as representations (think of accidental correlations). We agree, but note that the correlations between, for example, specific brain states and color perception look to fall onto the intuitively acceptable side of such a divide. (Clark and Toribio 1994, p. 417)

A proponent of this kind of argument will agree that causal covariation, taken alone, is not sufficient for representation. However, if this causal covariation occurs in a biological organ (like the brain) and has proven evolutionarily beneficial (has been selected for by the forces of natural selection), then it should be described in terms of representation.

One could, for example, argue for a teleosemantic account, upon which representation is thought to occur when there is causal covariation which has been selected for by evolution because it performs a fitness enhancing role (Dretske 1988; Millikan 1984). On this kind of account, we determine the representational function of a given mechanism by averting to its evolutionary history. Neural states which co-vary with a given *x* would be taken to represent *x* because they were selected for by evolution to respond to it. If the neural states happen to co-vary with *y*, where *y* is a non-natural stimulus introduced in the lab, then the neural states will misrepresent because natural selection did not select them for signalling *y*. Teleosemantics requires ascribing representation by determining the function of a given mechanism, with this function determined in turn by considering what the mechanism itself was selected to do by the forces of natural selection. It could therefore be argued that covariation between neural processing is representational because it has been selected for by the biological forces of natural selection.

Buttressing the concepts of “prediction-signal” and “error-signal” with teleosemantics, however, is not going to help these concepts pass the job-description challenge. Both prediction and error signals are concerned only with proximal conditions, and the functioning of both can therefore be adequately accounted for without invoking the concept “representation”. Consequently, applying teleosemantics to this particular example will not help with the job-description challenge, because teleosemantics is concerned primarily with the content of a given representation. Applying teleosemantics to a given mechanism involves discerning when representation and mis-representation occur. As such, teleosemantics is only applicable to scenarios in which the concept of “representation” has already been applied—the application of teleosemantics to a mechanism requires the assumption that a given instance of causal covariation is representational, and thereafter attempts to naturalise the particular content of that representation. The theory will not, therefore, help one in determining whether or not a given mechanism deserves to be described in terms of representation to begin with. In short, the theory of teleosemantics is only applicable once a given mechanism has already passed the job-description challenge. Consequently, teleosemantics cannot be used in an argument for the claim that instances of causal covariation pass the job-description challenge (cf. Hutto and Myin 2013).

In sum, the concepts “prediction-signal” and “error-signal” should be understood to involve mere causal correlation between neural processes. Causal correlation should not be considered sufficient for representation because it fails the job-description challenge. We do not gain any extra explanatory purchase by treating the causal covariation entailed by “prediction-signals” and “error-signals” in terms of representation. Although one can buttress causal correlation with concepts from evolutionary biology, doing so will not help with the job-description challenge. Therefore, I conclude that we can satisfactorily account for the role of prediction and error signals within the brain by conceiving of them in terms of mere causal mediation.

### “Priors”, “likelihoods”, and “posterior probability” are non-representational

Having outlined Orlandi’s argument that prediction and error signals fail the job-description challenge, I am now going to explain why Orlandi thinks that the concepts “prior” and “likelihood” also fail the job-description challenge. Orlandi argues that these concepts are best understood as referring to non-representational biases present in the neuronal system:

Understanding perceptual priors, hyperpriors and likelihoods as biases means thinking that, as a result of repeated exposure to the structure of the world in the evolutionary past *and* in the present, the perceptual system is skewed to treat certain stimuli in a certain way. (Orlandi 2015, p. 25, *italics in original*)

She argues that theorists are tempted to explain biases in terms of representation largely because they are in the grips of the traditional cognitivist idea that perception is to be understood in terms of internal inferential transitions between premises and conclusions in some kind of *language of thought* (cf. Ramsey 2009). Orlandi argues, however, that biases are more realistically understood to fulfil “the simple function of marking a hypothesis as more or less probable. They are like valves. They skew the brain toward certain neuronal arrangements” (Orlandi 2015, p. 25).

Consider the water fountain in my back garden. This fountain is made-up of three parts: a small bowl at the top (the mouth), a large bowl at the bottom, and a pumping mechanism which connects the bottom bowl to the top. The pump plays a biasing role within the fountain by ensuring that the vast majority of water in the fountain stays pooled in the bottom bowl, with only a small amount being pumped back up to the fountain top at any given time. We would not be at all tempted to ascribe the concept of “representation” to the functioning of this pump, and this is presumably because the ascription of representation to this pump fails the job-description challenge—describing the pump’s biasing role in terms of representation does not provide one with any explanatory benefits over and above those one would accrue by simply treating it as a mere non-representational bias in a water fountain system.

Orlandi contends that a similar conclusion should be drawn in the case of the PP concepts “prior” and “likelihood”. She argues that these concepts should be taken to refer to certain biases within a neuronal system, and that one should not treat these biases in terms of representation because there is no explanatory benefit in doing so. Consequently, Orlandi concludes that the concepts “prior” and “likelihood” are better understood as referring to mechanisms which pre-dispose brains to configure themselves into specific organisational patterns in response to environmental stimulation. Once more, the argument I am presenting here is not that biases cannot be understood in terms of representation. Rather, it is that their functioning can be understood entirely without invoking representation, and that treating them in representational terms is not explanatorily beneficial. Biases do not pass the job-description challenge, and so we have no reason to treat them in terms of representation. Therefore, I conclude that the PP concepts “prior” and “likelihood” should be taken to describe non-representational biasing processes occurring within the neural system.^4^

Consider, finally, the concept “posterior probability”. Although Orlandi argues for a non-representational stance toward the processes underlying PP, she concludes that the results of this processing (the resulting ‘winning hypothesis’, which is the hypothesis with the highest overall posterior probability) do deserve to be described in representational terms. Orlandi arrives at this conclusion because she thinks that the winning hypothesis fulfils the three conditions which she takes to be both necessary and sufficient for the ascription of “representation”:

[R]epresentations are only those performance-guiding structures that are de-coupled from their causes, where this fact materialises in their standing for distal or absent conditions. (Orlandi 2014, p. 133).

Orlandi claims that the winning hypothesis is concerned with distal conditions because it is formulated on the basis of sensory information received by the brain (photons, sound-waves, and so on) and yet is itself about things beyond brain-based sensory receptors (such as cats and leopards). She argues that the winning hypothesis is de-coupleable from its environmental causes because it can be deployed even in the absence of environmental causes. Finally, she argues that the winning hypothesis deserves to be treated in terms of representation because it is used by the brain to reason with and plan action. Consequently, according to Orlandi, although PP processing itself does not deserve a representational status the result of this processing does. I am inclined to reject Orlandi’s claim because I believe that it begs-the-question on two crucial points: (1) its *presumes *that cognition is concerned with distal, and not proximal, states-of-affairs; and, (2) it rests on an *assumption* of the problematic *representation demarcation thesis.*

#### Does cognition concern proximal or distal states of affairs?

Cognitivist theorists tend to assume that, in resolving a given cognitive task, the organism is restricted to the use of information and resources contained within the brain. Indeed, it is primarily for this reason that cognitivists invoke the concept of “representation” within their explanations at all: environmental input alone is generally considered too impoverished to explain successful cognition, and yet organisms do nevertheless successfully cognise. Cognitivists typically assert that the environmental poverty of stimulus for a given cognitive task is ameliorated via the presence of brain-based representations (Chomsky 1959; Fodor 1975; Marr 1982). Thus, “representation” is invoked by cognitivists in order to resolve a problem which only arises if one assumes that cognition is brain-bound. Orlandi’s claim that the PP ‘winning hypothesis’ is concerned with distal states of affairs, and her claim that it is de-coupleable from its causes, can only be made if one makes the prior cognitivist *assumption* that cognition is brain-bound: if cognition is brain-bound, then it follows that it will be concerned with distal states of affairs (events beyond the brain) and that internal cognitive states will be de-coupleable from their causes.

This cognitivist view of the mind can, however, be rejected. Proponents of enactive and ecological approaches toward mind, for example, deny that it is brain-bound. Rather, they claim that mind is constituted by the brain, the body, and the environment. Theorists working within these research traditions argue that an emphasis on the *performative *and *temporally extended *aspects of cognition will lead one to the realisation that there is no poverty of the stimulus in most cognitive domains (Anderson 2014; Barrett 2011; Chemero 2009; Gibson 1979; Hutto and Myin 2013; Thompson 2007). If there is no poverty of the stimulus in a given cognitive domain, then representation should be rejected in that domain because it is posited as the solution to a non-existent poverty of stimulus problem.

If one adopts such an enactive/ecological view of mind and applies it to the PP framework, the predictive brain can be considered to act as the categorical basis for cognition by enabling direct personal level cognitive contact with an organism’s environment (Anderson and Chemero 2013; Bruineberg and Rietveld 2014; Downey 2017; cf. McDowell 1994). If the environment is directly cognised, then cognition itself is concerned with proximal states of affairs and so should not be described in terms of representation. Furthermore, such instances of direct cognition cannot be de-coupled from their environmental causes (or, to be more precise, such instances cannot be de-coupled from their causes in a manner which necessitates the positing of representation).^5^ Therefore, if one adopts PP within an overall enactive or ecological approach to mind, the ‘winning hypothesis’ will be considered to enable direct cognitive contact with the organism’s environment and so should not be described in terms of representation. It will be taken to concern proximal states of affairs and it will not be understood as de-coupleable from its environmental causes.

#### Thought, action planning, and the ‘representation demarcation thesis’ 

This leaves us with Orlandi’s final condition, upon which the ‘winning hypothesis’ is described in terms of representation because it is used for thought and the planning of action. This conclusion is implausible because it appears to be based upon what Ramsey (2015) has labelled the “representation demarcation thesis” (RDT). Ramsey defines RDT as “the view that cognitive processes necessarily involve inner representations and cognitive theories must thereby be about the representational states and processes” (2015, p. 4). Orlandi’s final condition for ascribing a representational status to the winning hypothesis appears to be based on acceptance of a version of the RDT because, by her reasoning, it is a conceptual truth that any states which involve the planning and execution of action are representational states.

Ramsey provides three arguments against RDT. The first reason he provides for rejecting RDT is that it requires a conceptualisation of cognition which is arrived at by largely a priori means. Obviously, whether or not a given instance of cognition is to be understood in terms of representation should primarily be an empirical matter. However, by defining cognition in terms of representation (as RDT does), one guarantees that no instance of cognition will ever be non-representational. Either we must find a representational explanation of the cognitive activity in question, or, it will not count as a cognitive activity at all. Ramsey thus rejects RDT because it requires cognitive science accept a priori constraints on its domain of study and he thinks that no serious science should accept such constraints. His second reason for rejecting RDT is that RDT undermines the empirical status of the representational theory of mind. “Representation” is proposed as a theoretical posit which is supposed to play an empirical role in providing an empirical explanation of cognition. If, however, one accepts RDT, the cognitivist research programme within cognitive science no longer looks to be empirical in nature. Representation is not being posited for empirical reasons, because it plays an important role in an empirical theory of cognition. Rather, it is proposed for conceptual reasons, because it is a priori assumed that any empirical theory of cognition must be a theory of representation. Representation thereby becomes an unfalsifiable theoretical posit, and so the cognitivist research programme loses its empirical credentials. Consequently, Ramsey’s second reason for rejecting RDT is that it requires an unscientific approach to cognitive science. Finally, Ramsey rejects RDT because it encourages a wildly deflationary understanding of representation, such that even mere causal mediation or correlation is considered to be sufficient for representation (cf. Sect. 2.1). Aside from making the concept of representation itself almost vacuous, Ramsey concludes that deflationary accounts of representation can in fact hinder our investigation and resultant understanding of cognitive systems and so should be rejected.

Thus, although Orlandi does conclude that the ‘winning hypothesis’ should be understood in terms of representation, I have argued that this conclusion is not warranted because it is reliant upon the prior assumptions of: a cognitivist view of mind (upon which cognition is taken to be brain-bound); and, the RDT. Enactive and ecological versions of PP can accept that the ‘winning hypothesis’ forms the categorical basis of personal level cognition without thereby taking it be concerned with distal events or to be de-coupleable from its causes. Similarly, although such accounts could agree that the ‘winning hypothesis’ is causally implicated in guiding and executing action, concluding that it deserves a representational status on this basis alone is misguided, because reliant on acceptance of RDT. Therefore, the concept of “posterior probability” can satisfactorily be explained within PP accounts without thereby accepting representation.

At this point, we have arrived at a non-representational version of PP. “Prediction-signal”, “error-signal”, “prior”, “likelihood”, and “posterior probability” have all been described in non-representational terms. Importantly, no explanatorily beneficial insight was lost by describing these concepts in such non-representational terms. Having explained how the key PP posits can be understood in non-representational terms, I will now turn to explaining how even PP explanations which make indispensable use of representation can be accommodated within an eliminativist framework. I will argue that such representational posits are indispensable for epistemological, and not metaphysical, reasons.

## Fictionalism about the indispensable representational posits of PP

PP theorists are likely to object to the argument outlined above, because much of the work carried out within the PP research paradigm *requires* representation—even though we can provide a non-representational construal of PP’s key posits, much of the literature on PP simply cannot be made sense of without invoking the concept “representation”. The following quote, from Andy Clark, is representative of this general attitude:

Could we perhaps have told our story in entirely non-representational terms? One should always be aware of sweeping assertions about what might, one day, be explanatorily possible! But as things stand, I simply do not see how this is to be achieved. (Clark 2016, p. 293).

I am willing to concede that there are some PP explanations which do indispensably require the concept of representation. However, I will argue that, even when the concept is indispensable, we are not required to renege on the eliminativist conclusion of the previous section. My reason for making this claim is that I think representation is explanatorily indispensable in *some* PP explanations for *epistemological*, and not *metaphysical*, reasons. In this section I am going to apply a *make-believe approach* to scientific models to PP, and thereby explain how representational posits could be epistemologically indispensable to working scientists and yet metaphysically non-existent. I will conclude that representation can in some instances play an epistemologically indispensable role in PP models, but I will not thereby conclude that in such cases representation metaphysically exists.

### Motivating fictionalism

When concerned with ontological matters, there are (roughly) three attitudes one can take toward the entity in question:^6^

1. *Realismx* exists.
2. *Fictionalismx* does not exist. However, it plays a useful explanatory role and so can be treated as if it exists.
3. *Eliminativismx* does not exist.

Fictionalism about a given posit is often adopted by theorists when there is reason to believe the posit is ontologically suspect, and yet the posit itself is explanatorily indispensable. It is, for example, a popular position within debates about the metaphysics of modality and mathematics. In the case of mathematics, although it is difficult to envisage everyday human practice that does not make reference to mathematical objects, mathematical objects themselves are ontologically suspect (because they do not appear to exist in the same manner as spatio-temporal objects). Adopting fictionalism about mathematical entities enables one to keep them within one’s ontology whilst avoiding having to explain how they could causally interact with physical objects.

We have seen that the key posits of PP can all be explained without accepting or requiring representation. Nevertheless, it is arguable that much work on PP makes indispensable use of representational content (Clark 2015a, b, 2016; Gladziejewski 2016; Hohwy 2013, ch. 8; Orlandi 2014, 2015; Rescorla 2015; Seth 2014). Fictionalism is adopted when theorists both: make indispensable use of a concept; and, the concept is metaphysically problematic. PP explanations may sometimes make indispensable use of representation. However, representation is not necessitated within the framework and, moreover, rejection of the concept allows one to avoid many of the problems realism about sub-personal representation brings with it (Bennett and Hacker 2003; Hutto and Myin 2013). Consequently, in the present circumstances fictionalism about the indispensable representational posits of PP is well-motivated.^7^

### ‘Models as make-believe’ and predictive processing 

A number of theorists have recently applied Kendall Walton’s theory of pretence to the case of fictionalism about scientific models. They have argued that many scientific models provide theorists with prescriptions to imagine certain scenarios, and that the results of these imaginings allow for predictive and explanatory utility without committing theorists to the literal existence of each posit in their model. In this sub-section I introduce and motivate this approach by outlining Adam Toon’s work on the topic. Then I draw on the work of Arnon Levy, and explain how the application of representations to the brain (at least in the case of predictive processing) can be shown to be nothing more than metaphorical talk which is used by cognitive scientists as an instrumentally useful tool. I conclude that the indispensability of representation for PP models does not require commitment to the literal existence of representations. It is wholly compatible with the practice of PP that cognitive scientists are using representational talk only as a metaphorical tool which allows them to keep track of real causal interactions within the brain.

#### ‘Mimesis as make-believe’ and scientific representation

Walton’s (1990) ‘mimesis as make-believe’ account of fiction^8^ has in recent times been applied to work on scientific models by a number of philosophers. According to Walton, works of fiction act as props in games of make-believe which prescribe certain imaginings we must undertake. A number of philosophers of science have argued that scientific models should similarly be considered props in scientists’ imaginative games of make-believe (Frigg 2010; Levy 2011; Toon 2012). These models serve to prescribe imaginings the scientist should make with regard to a given system, and they are thought to play an instrumental role in helping scientists understand and predict real causal changes in the world. This means that the models themselves do not accurately represent the world and that they are not supposed to. Rather, they are used by the scientist as aides that help her to understand what is actually happening in the world.

Walton argues that fictions should be seen as analogous to the sorts of make-believe games which are played by children. These games are often based on what Walton labels “generative principles”, which are the rules upon which the imaginative game is based. Consider the example of a child playing a game with a bubble machine in their garden. In this game the bubbles are imagined to be space-ships which have escaped from the Death Star, and they must make their way home without being destroyed. Given the rules of this imaginative game, a number of generative principles follow:

1. The bubble machine counts as the Death Star.
2. The bubbles generated count as escaping space-ships.
3. The limits of the back garden, signified by the fence, count as the limits of the Death Star’s reach.

From these principles a number of sub-principles obviously follow. For example, a corollary of (2) is that:

1. The larger the bubble, the bigger the spaceship is.
2. Bubbles which pop count as destroyed.
3. Bubbles which float over the garden fence count as having escaped.

Although the child is engaged in a game of imaginative make-believe, the things which happen in the game depend entirely on things which happen in the actual world. For example, the fate of a given space-ship depends entirely on the fate of a bubble in the actual world. Its fate cannot be decided by the whims of the child. Consequently, the child is able to accurately track events which occur in the real world. For instance, they can track how many bubbles float past the garden fence.

#### Levy’s ‘make-believe’ account of information in biology 

Levy (2011) argues that when biologists make use of the concept of (semantic) “information” they are engaged in a Walton-esque game of make-believe. Just as the child’s game is constrained by states of affairs in the real world so, according to Levy, the biologist’s use of the concept “information” is constrained by states of affairs in the world (at the biological level of description). He outlines three rules (about the features of biological systems which function as props in the information-in-biology fiction) which the biologist must (and does) adhere to (*ibid*., pp. 654–655):

1. *Directionality* information is transmitted from a sender to a receiver.
2. *Connecting variation* transmitted information will cause changes in the receiver (this allows us to gloss over causal events which occur between sender and receiver).
3. *Active versus passive distinction* this is a metaphor, which gets at the difference between the parts of the system which change and the parts that do not. The metaphor “active” refers to the changes of state in sender and receiver (of information), whilst the metaphor “passive” refers to the parts of the system which do not change (the information itself).

Levy does note that an argument similar to his own could be given about the use of information talk in neuroscience. However, he does not himself provide such an argument. In the next sub-section I will apply Levy’s three rules to the case of predictive processing.

### Applying Levy’s fictionalism to predictive processing

**Fig. 1:**
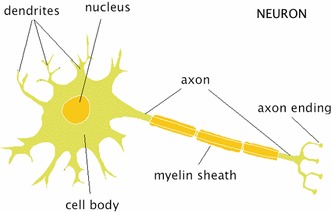
A Neuron (Boeree 2009)

I begin my application of Levy’s informational principles to the brain by considering the simplest component of the brain (generally) considered relevant for our ability to engage in cognitive activities—the neuron. A neuron is a cell which is composed of three parts: dendrites, soma (or cell body), and axon (Fig. 1).

Neurons both receive and pass on information in the form of electrical and chemical signals. Electrical and chemical signals are passed across the synaptic cleft (the gap between the axon of one neuron and the dendrite of another) and picked up by the dendrites. These chemical and electrical signals move into the soma of the neuron, and when they reach a certain concentration (the exact concentration can vary from cell to cell) an action potential will send electrical signals down the axon and cause the neuron to fire. When a neuron fires it passes electrical and chemical signals from its axon to the dendrites of other cells.^9^

At this neurophysiological level of description Levy’s three rules are satisfied. Directionality is satisfied because signals pass from one neuron to another (from the axon of one to the dendrites of another). Connecting variation is also satisfied, because the electrical and chemical constitution of the soma of the receiving neuron will change in response to the electrical and chemical signals received from the neuron which passed on the signal. Finally, the metaphorical distinction between active and passive is also satisfied. Although the electrical and chemical constitution of the two neurons will change, the information being transmitted remains the same. This information is constituted by informational signals and these remain unchanged whilst they pass across the synapse. At this level of description there is no need for the use of representational posits. Indeed, the only predicate in use which is remotely psychological is the term “signal”. However, this term refers to electrical and chemical properties and so can be seen as straightforwardly metaphorical. It can be translated into the language of chemistry and physics with no loss in explanatory power.

In order to consider a second model of neural processing, I will now ascend a few levels of description:^10^

**Fig. 2:**
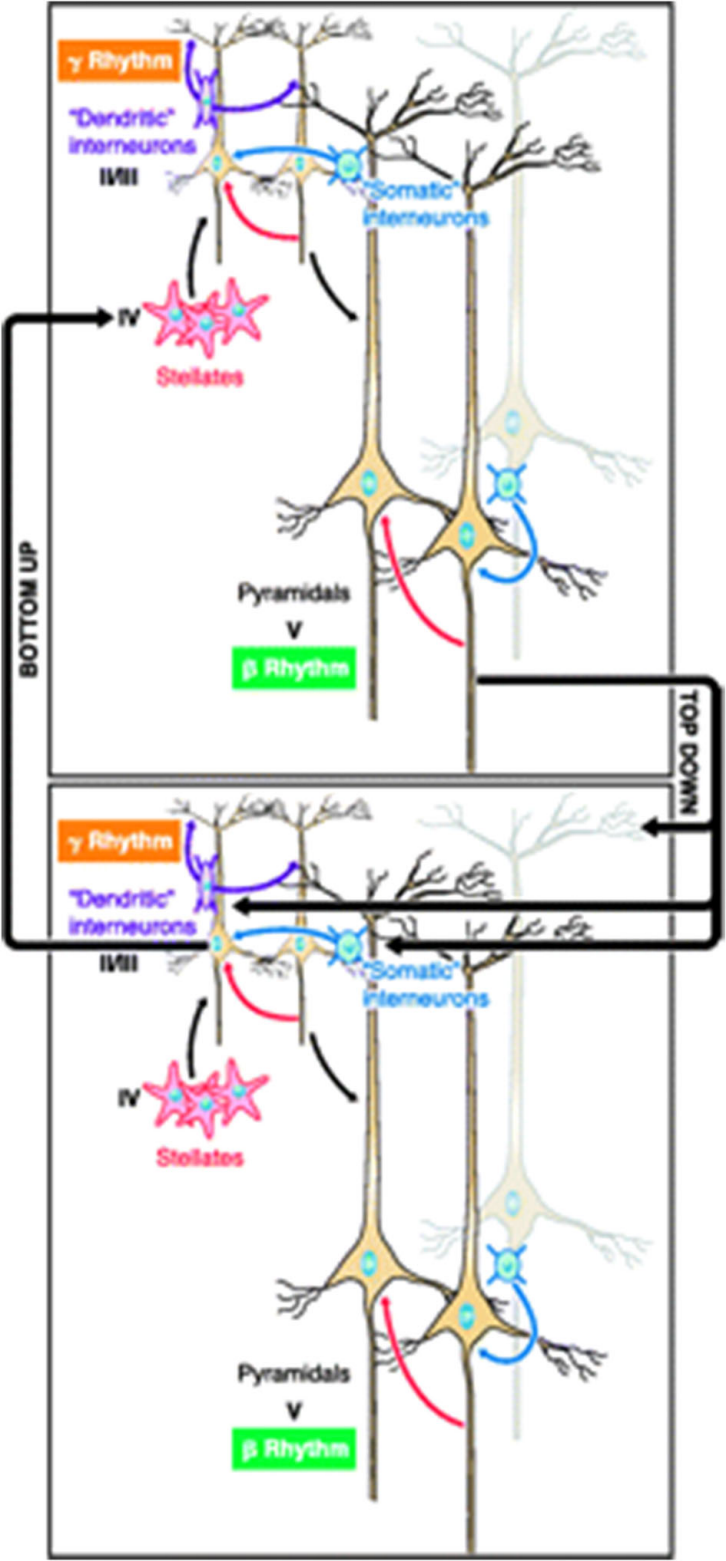
Top-down and bottom-up signalling between cognitive and sensory areas of the brain (Wang 2010, Fig. 19)

This diagram depicts the mechanism through which the passing and regulation of top-down and bottom-up signals occurs between cognitive (top box) and sensory (bottom box) areas of the brain.^11^ Neurons located in deep layers of the brain are represented in the lower half of each box. These neurons oscillate in the beta-range and are responsible for sending top-down signals to neurons located in superficial layers of the brain. Neurons in superficial layers are represented in the top half of each box. They oscillate in the gamma-range and send bottom-up signals to neurons in deep layers. The exact nature of the signals sent and received from both deep and superficial layers is dictated by a dynamical inter-play between beta- and gamma- range oscillations (Wang 2010, Fig. 19).

At this higher level of description, which makes reference to groups of neurons in different regions of the brain, Levy’s rules are still satisfied. Neurons in the bottom half of each box are designated as the senders of top-down signals whilst neurons in the top half of each box receive these signals. Similarly, neurons in the top half of each box are designated as the senders of bottom-up information whilst neurons in the bottom half are designated as receivers. Consequently, directionality is satisfied. Connection variation is also satisfied because the neurons (whether situated in deep or superficial layers of the brain) will change in response to the signal received. The nature of this change is more complicated than the previous example of the lone neuron. This is because the exact manner in which the neurons change is now dependent on an inter-play between top-down and bottom-up signalling. However, the fact that neurons will change state in response to the signals they receive remains transparent. Finally, the active and passive metaphor is still satisfied. Deep layer neurons are associated with beta-range oscillations, and superficial layer neurons with gamma-range oscillations. As such, the oscillations they rely on to transmit information and the type of information they transmit never changes, although the states of the neurons themselves will change. Although we have ascended to the level of many neurons which are situated in spatially separated parts of the brain, this description of brain processes is clearly firmly rooted in what we can observe and understand using the tools and framework of neurophysiology. If any psychological predicates are used at this level of explanation it is clear they are being used metaphorically.

**Fig. 3:**
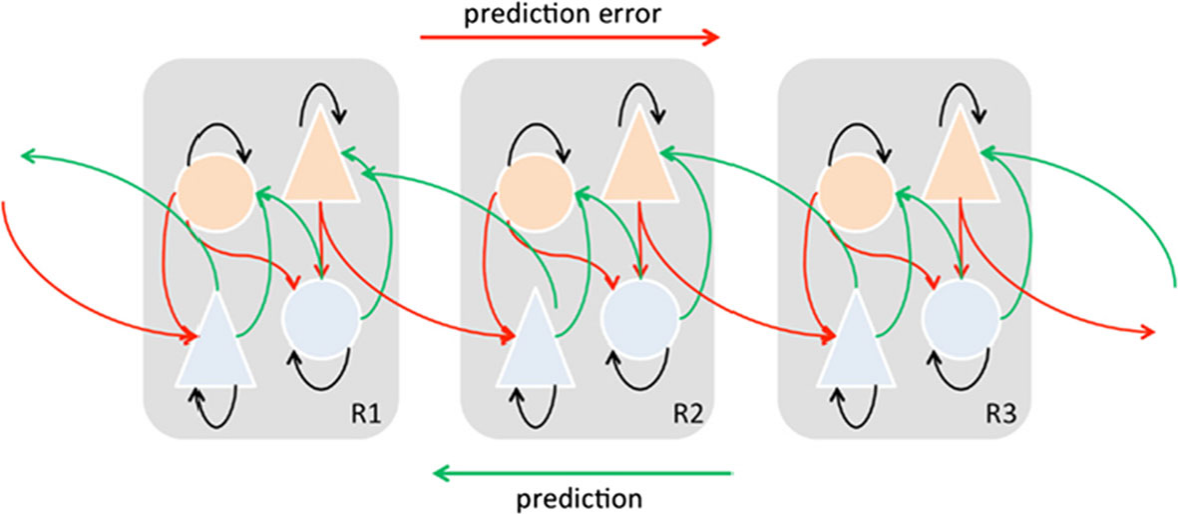
Predictive processing model of the brain (Seth et al. 2012, Fig. 3)

I will now consider a third diagram. This diagram is also concerned with top-down and bottom-up neural interactions. However, it is pitched at a slightly higher level of abstraction than the previous diagram:

This diagram is a representation of how the predictive processing paradigm conceives of the brain. R1 through to R3 represent different levels of the brain, from superficial (R1) to deep (R3). The green lines are the same top-down signals which are linked with beta-range oscillations that we encountered in Fig. 2. The PP theorists have added to the interpretation made in Fig. 2 by labelling the top-down signals “prediction-signals” and arguing that their role is to transmit predictions down the neural hierarchy. The red lines represent the bottom-up signals associated with gamma-range oscillations that were encountered in Fig. 2. Once more, the PP theorist has added to this interpretation by labelling the bottom-up signals “error-signals”. On the PP framework these error-signals feedback information that there has been a divergence between the predicted signal and the signal the neuron actually received. Prediction neurons are coloured blue and error neurons are coloured orange (Seth et al. 2012, Fig. 3).

Although this model makes use of a lot more representational idioms than any of the previous diagrams, it is obvious that Levy’s rules are still being satisfied. The exact same directionality noted in Fig. 2 is satisfied because top-down signals (now labelled “prediction-signals”) still transmit from deep to superficial layers. Similarly, bottom-up signals (now labelled “error-signals”) display the same directionality, going from superficial to deep layers. Connection variation is still satisfied because the type of signals sent (by neurons in both the deep and superficial layers) will depend on the type of signals they receive. However, the application of psychological predicates allows for a simplification of the exact relation between the top-down and bottom-up signals. By construing top-down and bottom-up signals as representational prediction and error signals respectively, the PP theorist is able to arrive at improved predictions and explanations about the exact nature of the relationship between signalling in the deep and superficial layers of the brain. Finally, the active/passive metaphor is still in place. The information transmitted stays the same throughout (it will either be a prediction-signal or an error-signal), with only the neurons themselves changing in response to the information received. In short, the exact same causal processes as those in the previous diagrams are being tracked. The only difference is that they are being tracked at a higher level of generality (Fig. 3).

Toon defines his ‘models as make-believe’ [MM] account as follows:

MM: M is a model representation if and only if M functions as a prop in a game of make-believe. (Toon 2012, p. 62)

I have argued that the PP paradigm adheres to the ‘brain-as-representational-Bayesian-machine’ game of make-believe. In this particular case the brain is the prop upon which the game of make-believe is predicated (upon which the various ‘generative principles’ are based). I studied three diagrams of neuronal processing. Each successive diagram represented the brain at a higher level of abstraction, and as the level of abstraction increased so did the use of representational idioms. However, every single diagram satisfied Levy’s three principles in the same manner. The exact same causal changes were modelled in each diagram with the only difference being that, as the models became more abstract, the causal changes were modelled in more generality. The neuroscientist is able to ascend from the level of individual neurons, and instead model interactions between groups of neurons (which may be spatially separated to a large degree), by introducing representational predicates. This practice allows the scientist to model real causal processes without having to concern themselves with the minutiae of detail which consideration of the brain as a whole would require. Rather than being seen as pointing to the metaphysical indispensability of brain-based representations, the introduction of representational idioms can be seen as nothing more than a metaphorical and fictional posit which allows the neuroscientist to keep track of real causal changes occurring at the neurobiological level. Treating the brain as if it is a representational prediction machine prescribes certain imaginative games, which both help and allow the neuroscientist to track real causal changes occurring within the brain at a greater level of generality.

## The spoils of war

In this paper I have argued that: the key posits of PP need not be described in terms of representation; and, PP accounts which make indispensable use of representation can be understood as doing so for epistemological reasons. If the forgoing is correct, within the PP framework, representation is either rendered entirely otiose or it is assigned a fictional status. This conclusion licenses an eliminativist conclusion about the metaphysical status of representation within PP. In this section, I am going to outline four benefits one arrives at by adopting the approach toward representation which I have sketched.

### Benefit one: a victory for the eliminativist

The most obvious consequence of the position I have sketched is that PP entails victory for the eliminativist in the ‘representation wars’. If the foregoing is correct, PP entails metaphysical eliminativism about sub-personal representation. This is a beneficial conclusion, because it allows for PP theorists to continue to provide accounts of perception, action, and cognition without facing the problem of having to naturalise sub-personal content. It is widely agreed that, as yet, no solution to this *hard problem of content *(Hutto and Myin 2013) has been found. By accepting metaphysical eliminativism, PP theorists can continue to investigate perception, action, and cognition without being reliant on an assumption (there is sub-personal representation) which is naturalistically suspect. Furthermore, by accepting a fictionalist approach toward PP’s indispensable representational posits, one can accept metaphysical eliminativism without thereby having to reject work in PP which is reliant upon representation. Realism about representation is primarily advocated because representation is taken to be an indispensable posit of cognitive science (Burge 2010). Adopting a fictionalist approach toward representational posits allows one to endorse metaphysical eliminativism about representation without requiring we disavow scientific explanations which make indispensable use of this concept. Consequently, the account I have outlined entails a victory for the eliminativist in the ‘representation wars’. Importantly, however, this particular position can accommodate extant empirical work within PP which makes indispensable use of representation whilst avoiding the problems invocation of this posit brings with it.

### Benefit two: fits with practise of cognitive science

A second benefit of this fictionalist approach to representation is that it fits with the everyday practice of cognitive science. My account is inspired by, and finds support from, the work of Dennett (1987). Dennett argues that representational idioms are used within cognitive science because they provide scientists with instrumentally useful metaphors:

Far from it being a mistake to attribute hemi-demi-proto-quasi-pseudo intentionality to the mereological parts of persons, it is precisely the enabling move that lets us see how on earth to get whole wonderful persons out of brute mechanical parts. That is a devilishly hard thing to imagine, and the poetic license granted by the intentional stance eases the task substantially. (Dennett 2009, pp. 88–89)

Frances Egan argues for a similar claim:

What we normally think of as representational contents—contents defined on distal objects and properties appropriate to the cognitive domain (what I have called ‘cognitive’ contents)—are not in the theory; they are in the explanatory gloss that accompanies the theory, where they are *used* to show that the theory addresses the phenomena for which we sought an explanation. The gloss allows us to see ourselves as solving problems, exercising rational capacities, occasionally making mistakes, and so on. It characterizes the computational process in ways congruent with our commonsense understanding of ourselves, ways that the theory itself eschews. (Egan 2013, p. 131, *italics in original*).

According to Egan, representational explanations in cognitive science provide a user-relative *cognitive gloss* on neuronal mechanisms which helps scientists to understand the workings of the mechanism in question.

The approach to sub-personal representation advocated by both Dennett and Egan is motivated by considerations about the everyday practice of cognitive science. The argument I have provided can be seen to support Dennett and Egan’s contention—if PP theorists do use the brain as a prop in the imaginative game of ‘brain-as-representational-Bayesian-machine’, then PP provides yet another example of cognitive science in which representational posits are in fact being used merely as instrumentally useful metaphors. Indeed, I think that PP provides a particularly strong example of such an instrumentalist approach toward representation. We have already seen that some theorists take PP to indispensably require representation only at the more abstract, system-wide level of explanation (Orlandi 2014, 2015; see also Clark 2015a, b, 2016). This observation would appear to be borne out, more generally, by looking closely at the actual work carried out within PP. In general, representational idioms are most pervasive when PP systems are being described in the abstract. However, when such systems and their implementation are described in greater detail, representational idioms tend to drop out of the resultant explanation. One prominent example, in this regard, is the discussion of the neural implementation of precision-weighting. Precision-weighting is often described in representational terms:

However, to optimally select the prediction errors...the brain has to estimate or encode their precision. Having done this, prediction errors can then be weighted by their precision, so that only precise information is accumulated and assimilated in high or deep hierarchical levels. (Kanai et al. 2015, p. 3)

The use of concepts and phrases such as “estimate”, “encode”, and “informational accumulation” naturally lend themselves to a representation-friendly interpretation. However, when the neural implementation of these representational terms is presented, it becomes clear that what we are discussing here is simply biological neural processing:

This broadcasting of precision-weighted prediction errors may rest on neuromodulatory gain control mechanisms at a synaptic level...This may explain why superficial pyramidal cells have so many synaptic gain control mechanisms such as N-methyl-D-aspartate (NMDA) receptors and classical neuromodulatory receptors like D1 dopamine receptors. (Kanai et al. 2015, p. 3)

Kanai et al. do themselves take this neurobiological processing to “correspond[] to a (Bayes-optimal) encoding of precision in terms of the excitability of neuronal populations reporting prediction errors” [*ibid*. p. 3]. However, it is not clear why this reading is required. Certainly, bio-chemical neuronal activity itself does not require a representational explanation. If precision-weighting can be equated with neuromodulation, then there does not appear to be any reason to accept a representational account of precision-weighting. It is thus typically the case that, when the neural details are specified, a representational explanation is no longer necessary.

I therefore take it to be wholly compatible with the practice of PP theorists that, when representational posits are considered indispensable, they are considered indispensable largely because theorists are engaged in a game of make-believe which treats brains as if they were representing. These games of make-believe are grounded on a number of generative principles which rely on real causal goings-on in the brain. Consequently, the metaphors themselves are not relevant to causal interactions in the brain, but rather only to our ability to understand and track these interactions. The practise of PP theorists therefore does not warrant taking the brain to be literally trafficking in representations. Rather, it warrants the weaker claim—that scientists (sometimes) use the brain as a prop in a game of pretence involving representations.^12^

### Benefit three: recommendations for the direction of future research

Aside from fitting well with the actual practice of theorists working on predictive processing, my account also has direct, practical implications for future research. If I am correct, then it follows that theorists need not concern themselves with finding specific neural implementations of the representational posits of their PP models. These models merely prescribe specific imaginings, and so it is no requirement of them that each representational aspect of the model be found to be isomorphic with some aspect of the world. If my fictionalist account is correct, then it will not be the case that failure to find a neural implementation of (representational aspects of) PP means that it “fails as a distinctive empirical account” (Clark 2013, p. 200). As such, theorists can continue to use representational posits in their theories. However, if they do make use of such posits, they should not dedicate their energies toward finding the neural implementation of these representational posits. PP theorists tend to take their explanations to require a mechanistic implementation of representation (Gladziejewski 2015, 2016; cf. Bechtel 2009). However, acceptance of my fictionalist account requires a turn away from such endeavours. Therefore, fictionalism about the representational posits of PP would have a practical effect on the direction of future research.^13^

### Benefit four: Sprevak’s IBE

A common argument against all fictionalist positions is that they are explanatorily unilluminating. In a recent survey concerning fictionalism about sub-personal representation, Sprevak (2013) highlights this as a problem any fictionalist position must face. Normally, when a scientific explanation works, the rule of inference to the best explanation (IBE) is invoked. The best explanation for the success of the scientific model is that it accurately describes the phenomenon in question. It seems to follow by IBE that if PP theorists cannot do their work without making reference to “representation”, then sub-personal representations exist. Sprevak notes that this problem is particularly pressing in present circumstances because the fictionalist must “find reasons for rejecting IBE in the case of neural representations that do not apply to other areas of cognitive science where IBE is employed” (2013, p. 557). My account avoids this IBE problem because in the particular case at issue we have specific, local, reasons for blocking individual attempts (by representationalists) to accept an IBE.

Compare the two competing claims:

- IBE: the brain is a representational Bayesian computer.
- Fictionalism: the brain is not a representational Bayesian computer. However, its functioning is comparable in certain important respects to that of a representational Bayesian computer, and so it can usefully be treated *as if* it were one.

Speaking explanatorily, (a) and (b) are equivalent because they both allow us to explain the success of representational PP. Theorists tend to accept option (a) because an IBE provides the simplest explanation of why representational PP explanations work. However, we have seen in this paper that (a) is problematic for theoretical and philosophical reasons. Acceptance of (a) is problematic because: (1) PP posits do not require a representational explanation [Sect. 2, *this paper*], (2) it invokes the extremely thorny *hard problem of content *(Hutto and Myin 2013), and, (3) it *assumes *that the epistemological indispensability of representation entails their metaphysical existence (i.e. it ignores the possibility that representations could be merely instrumentally useful fictional posits). I have argued that (b) provides a better explanation of the success of representational PP precisely because it can account for the success of such explanations whilst avoiding the problems metaphysical realism about sub-personal representation brings.

I am therefore not arguing that we should not use IBE in cognitive science. Nor am I endorsing the claim that we should be sceptical of IBE across the board. Rather, I am claiming that, in this particular instance, we have no need to use IBE. The reason for this is that a fictionalist explanation of PP representation is better than a literalist one. Applying fictionalism does not require an abandonment of IBE. It instead involves proper application of IBE, because it involves making proper evaluations of what a best explanation actually entails.^14^

## Conclusion

I began this paper by mentioning Andy Clark’s recent claim that the concept of “representation” used within the predictive processing framework requires an end to the ‘representation wars’. According to Clark, the concept is so far removed from traditional conceptions of representation that it dissolves the problems upon which the ‘representation wars’ were predicated. I have argued that Clark is correct to think that PP requires an end to the ‘representation wars’. However, I have concluded that PP requires a cessation of hostilities because it entails an eliminativist victory. In Sect. 2, I outlined the key posits of predictive processing and explained how each could be adequately accounted for without invoking or requiring the concept of “representation”. Then, in Sect. 3, I explained how metaphysical eliminativism could be made compatible with even predictive processing explanations which make indispensable use of representation, because representation is indispensable in these explanations for epistemological (not metaphysical) reasons. I explained how representational posits could play an indispensable epistemological role within PP explanations without thereby requiring that representations metaphysically exist in such systems. Finally, in Sect. 4, I highlighted four benefits one accrues by endorsing my approach toward representation in predictive processing: one avoids the problem of having to naturalise sub-personal representation whilst still being able to account for empirical work carried out within PP; my account is consistent with, and indeed influenced by, actual empirical practice; my account provides guidance for future research; and, my account provides (the beginnings of) a response to Sprevak’s IBE problem for fictionalist approaches toward sub-personal representation. I therefore conclude that, by combining extant eliminativist accounts with a fictionalist approach, we arrive at an attractive conception of predictive processing which is both empirically beneficial and metaphysically anodyne.

## Footnotes

1 Given that the ‘representation wars’ are chiefly concerned with whether perception, action, and non-linguistic cognition require a representational or non-representational explanation, my argument is that predictive processing accounts of perception, action, and non-linguistic cognition do not require representational explanations. I am not making the stronger claim that no predictive processing explanations require representation. Predictive processing explanations of personal level linguistically-mediated cognition, for example, would most likely be best explained in terms of representation.

2 My thanks to both an anonymous referee and Roger Clarke for helping me to clarify the mathematical details in this paragraph.

3 It might be objected that prediction and error signals are ultimately concerned with *distal, *and not *proximal*, conditions. I defend the claim that they be understood as concerned only with proximal conditions later in the paper (Sect. 2.3.1).

4 For a more detailed explanation of what non-representational biases are, and an explanation of how they can be empirically investigated, the interested reader can consult (Anderson 2014; Anderson and Finlay 2014; Orlandi 2014, 2015).

5 For extended defences of the claim that direct cognition does not require, and should not be described in terms of, representation, the interested reader can consult (Anderson 2014; Chemero 2009).

6 This taxonomy is grossly over-simplified [see (Chakravartty 2010, ch. 1), for a more realistic taxonomy].

7 Henceforth, when I mention or discuss the representational posits of PP, I should be understood to be referring to PP accounts which make indispensable use of representation. If a PP account can be straight forwardly accepted without representation, there will obviously be no need to provide a fictionalist account of its representational posits (because there are none).

8 Note, applying Walton’s account to scientific models does not require taking a stance on the nature of fiction itself—“I do not want to defend Walton’s characterisation of fiction here. As I see it, we may remain neutral on this point. As a result, the make-believe view does not commit us to the claim that models are works of fiction.” (Toon 2012, p. 70).

9 This is a simplified description of a typical neuron.

10 For a comprehensive review of explanations of how to get from a description of a single neuron to a description of interactions between groups of neurons in different parts of the brain, the reader should consult Wang (2010).

11 By speaking in this manner I mean only that these areas are causally implicated in cognitive and perceptual processing.

12 One might object to this point as follows: (1) PP developed out of the machine learning literature. (2) The concept “representation” plays an extremely specific and nuanced role in this literature. Therefore, (3) The concept “representation” is plausibly understood as being used by PP theorists in exactly the same manner it is used within machine learning. Consequently, the practice of PP does not license the claim that “representation” plays the role of tracking mere causal changes within the brain. Spatial constraints require that I do not fully engage with this objection here and so, for present purposes, I suggest that those who find this objection persuasive understand my argument as a conditional: *If *PP theorists use the concept “representation” to track causal changes within the brain, *then *these representational posits should be assigned the metaphysical status of a fictional entity. It is hard to determine the exact use to which “representation” is put by every single individual theorist (cf. ft. 13). However, I contend that enough theorists use the concept in a manner which is best understood as involving the tracking of mere causal changes [e.g. (Clark 2016; Hohwy 2013; Kanai et al. 2015; Seth 2014)] that the initial premise of my conditional argument is substantive and its adoption is motivated. My thanks to an anonymous reviewer for both: bringing this potential objection to my attention; and, suggesting the adoption of the above conditional argument in order to avoid it.

13 It may be wondered how fictionalism about the representational posits of PP could be compatible with the practise of cognitive science whilst also providing guidance for future research which goes against common practice. To understand why these two conclusions are not incompatible, it helps to introduce the two different types of fictionalism: *hermeneutic* and *revolutionary*. Hermeneutic fictionalists think that, when engaged in the discourse in question, people already (tacitly) assume that they are speaking in a fictional context. Revolutionary fictionalists argue that, although people who engage in the discourse in question do take themselves to speak literally, we should revolutionise the discourse by encouraging people to stop speaking literally and start taking their discourse to be metaphorical. It is an empirical question which of these approaches to fictionalism correctly describe the practice of theorists working on PP, and in any case I suspect that the answer will vary from person to person (and, indeed, possibly from case to case). In light of this distinction between the two types of fictionalism, I suggest that benefit two be seen as applicable to those who already (if only implicitly) take the representational discourse to be a useful heuristic, whereas benefit three should be taken to apply to those who take the present discourse to require ontological commitment to representation.

14 Of course, more needs to be said on this point. Here, I aim only to motivate the idea that this account gives us good initial (defeasible) reasons to think we avoid the IBE problem.
